# Polydopamine-Bi_2_WO_6_-Decorated Gauzes as Dual-Functional Membranes for Solar Steam Generation and Photocatalytic Degradation Applications

**DOI:** 10.3390/polym13244335

**Published:** 2021-12-10

**Authors:** Yea-Chin Wang, Chi-Jung Chang, Chih-Feng Huang, Hao-Cheng Zhang, Chun-Wen Kang

**Affiliations:** 1 Department of Chemical Engineering, Feng Chia University, 100, Wenhwa Road, Seatwen, Taichung 40724, Taiwan; wyj05@ms17.hinet.net (Y.-C.W.); ss0911445818@gmail.com (H.-C.Z.); wainss9407278@gmail.com (C.-W.K.); 2 Department of Chemical Engineering, i-Center for Advanced Science and Technology (ICAST), National Chung Hsing University, Eng Bld 3, 250 Kuo Kuang Road, Taichung 40227, Taiwan; HuangCF@dragon.nchu.edu.tw

**Keywords:** Bi_2_WO_6_, polydopamine, photocatalytic degradation, solar steam generation

## Abstract

The dual-functional Bi_2_WO_6_/polydopamine (PDA)-modified gauze membrane has been developed for applications in photocatalytic degradation and solar steam generation. Two types of membrane were prepared by changing the growth sequence of Bi_2_WO_6_ nanomaterials and PDA on gauze substrates. The spatial distribution of Bi_2_WO_6_ and polydopamine has a great influence on light absorption, photocatalytic degradation, and solar steam generation performances. Bi_2_WO_6_ photocatalysts can absorb short-wavelength light for the photocatalytic decoloration of organic dyes. The photothermal polydopamine can convert light into heat for water evaporation. Besides, the gauze substrate provides water transport channels to facilitate water evaporation. The morphology, surface chemistry, and optical properties of Bi_2_WO_6_-PDA modified gauzes were characterized by scanning electron microscopy, transmission electron microscopy, X-ray photoelectron spectroscopy, and diffuse reflectance spectra. The photothermal properties, wetting properties, and solar steam generation rates of the composite films were also studied. Degradation of 96% of indigo carmine was achieved after being irradiated for 120 min in the presence of G/PDA/BWN_P_. The water evaporation rates of the G/BWP/PDA sample under the irradiation of an Xe lamp (light intensity = 1000 W/m^2^) reached 1.94 kg·m^−2^·h^−1^.

## 1. Introduction

Developing practical approaches to obtain clean water and eliminate pollutants is an important issue for our society because of the increasing world population [1] and water contamination caused by various industrial production processes. Besides, since solar energy is a sustainable and renewable energy source, light energy has been widely utilized in photovoltaic [2,3], photothermal [4,5,6], and photocatalytic [7,8] applications. Solar energy-driven water production is significant for people living in remote areas without electricity supply [9]. Solar steam generation is a promising process to produce clean water from seawater [10,11] or contaminated water sources [12,13]. Meanwhile, photocatalysts can be applied to the photocatalytic degradation of organic pollutants or the reduction of heavy metal ions [14,15]. Pollutants can also be degraded in the presence of heat (thermocatalysis) or light (photocatalysis, photoactivation and photothermocatalysis) [16]. Therefore, the development of dual-functional membranes for water treatment by the combined solar steam generation and photocatalytic degradation approaches is worth studying. Yang et al. [17] developed a novel dual-functional water-treatment system by integrating photothermal Ti_3_C_2_T*_x_*, La_0.5_Sr_0.5_CoO_3_ photocatalysts, and polyvinyl alcohol/chitosan hydrogels. The system can achieve high photothermal conversion performance and photodegradation activity. Jin et al. [18] reported that the Pt/Au/TiO_2_ NP-wood carbon composite materials exhibited outstanding interfacial solar steam generation property and photocatalytic degradation activity toward tetracycline. Ding et al. [19] reported a large-scale outdoor solar conversion device that was constructed to simultaneously harvest freshwater from contaminated water and degrade organic dyes in the source water. The daily freshwater production rate is 5.7 kg m^−2^. This demonstrated the potential of the membrane for the production of potable water and the treatment of contaminated water using one device.

The combination of polymer and inorganic materials was useful for improving various properties, such as solar steam generation, moisture blocking, photocatalytic degradation, photoconductive, and gas sensing [20,21,22,23,24,25,26] performance. Polydopamine (PDA) exhibits excellent adhesion, biocompatibility, hydrophilicity, and photothermal conversion properties [27]. It has been used for various applications, such as adsorbents for heavy metals ions [28,29], photothermal cancer therapy [30,31,32], and solar steam generation [33]. PDA was used in the preparation of some polymer/inorganic composite photocatalysts. PDA modification was effective in improving the activity of g-C3N4, BiOBr, BiOCl, ZnO, BiVO_4_, and TiO_2_-based composite photocatalysts that can be used for photocatalytic degradation, photocatalytic CO_2_ reduction, and removal of hexavalent chromium [34,35,36,37,38]. Furthermore, polydopamine-inorganic composites were loaded on fabrics for catalysis applications. Liu et al. reported the preparation and properties of flexible Ag/AgCl/polydopamine/cotton fabric-based photocatalysts [39]. Wang and coworkers studied the UV protection, antimicrobial activity, and photocatalysis property of CuO/BiVO_4_-polydopamine and ZnO/polydopamine-modified cotton fabrics [40,41].

In recent years, Bi-based photocatalysts have attracted lots of attention, due to their tunable morphology, unique electronic band structure, and excellent chemical stability [42,43,44]. Bismuth tungstate (Bi_2_WO_6_) photocatalyst was developed to remove organic pollutions due to its advantages, such as tunable morphology, excellent stability, low cost, and high photocatalytic activity [45,46]. The activity of photocatalysts can be improved by introducing conductive polymer, carbon nanotube, carbon dots, and metal substrate [47,48,49,50]. Since it is difficult for Bi_2_WO_6_ photocatalysts to be separated and collected for repeated operation, the growth of Bi_2_WO_6_ nanomaterial on a porous substrate to make the immobilized photocatalyst is worth developing. Bi_2_WO_6_ photocatalysts have been coated on different supporting materials such as indium-tin-oxide glass [51], stainless steel [52,53], and polyester fabric [54,55] to make immobilized photocatalysts, exhibiting some advantages such as enhanced photocatalytic activity, self-cleaning properties, and recyclability. Indigo is a widely used dye in the textile industry for the dyeing of blue jeans and other blue denim products [56]. Its strong intermolecular hydrogen bonding leads to a high melting point and poor solubility. Indigo carmine (IC) exhibits better solubility. However, it is carcinogenic and can cause severe health problems [57,58]. In this work, we studied the decolorization of an IC dye to evaluate the degradation by various photocatalysts. The utilization of gauze as the porous substrate to support the functional polydopamine and Bi_2_WO_6_ in this work has some advantages. Its interconnected pore structure can provide water transport channels for photothermal evaporation. Besides, it was reported that a rough surface could enhance light trapping of incident solar light by multi-scattering [59,60]. The large specific surface area of microporous gauze also increases the contact area between the loaded Bi_2_WO_6_ and pollutants in wastewater, leading to enhanced water treatment capacity.

Considering the high activity of Bi_2_WO_6_, together with the broad optical absorption and excellent photothermal conversion characteristics of PDA, two types of PDA-Bi_2_WO_6_-gauze-based dual-functional membranes, G/BWP/PDA and G/PDA/BWN_P_, were developed by the sequential growth of Bi_2_WO_6_ nanomaterials and PDA on gauze substrates through microwave-assisted hydrothermal and self-polymerization methods. The effects of the spatial distribution of Bi_2_WO_6_ and polydopamine on the light absorption properties, photocatalytic degradation, and solar steam generation performances of these films were studied.

## 2. Experimental

### 2.1. Nomenclature

G, G/PDA, and G/PDA/BWNp mean the gauze substrate, gauze/polydopamine, and gauze/polydopamine/Bi_2_WO_6_ nanoparticles samples, respectively. G/BWP and G/BWP/PDA indicate the gauze/Bi_2_WO_6_ nanoplates and gauze/Bi_2_WO_6_ nanoplates/polydopamine samples, respectively.

### 2.2. Preparation of Dual-Functional Membrane

The non-sterile non-woven rayon/polyester gauze is provided by YASCO Enterprise Corp. Two types of dual-functional film were developed by the sequential growth of Bi_2_WO_6_ nanomaterials and PDA on gauze substrates for applications in photocatalytic degradation and solar steam generation in this study.

**Figure 1:**
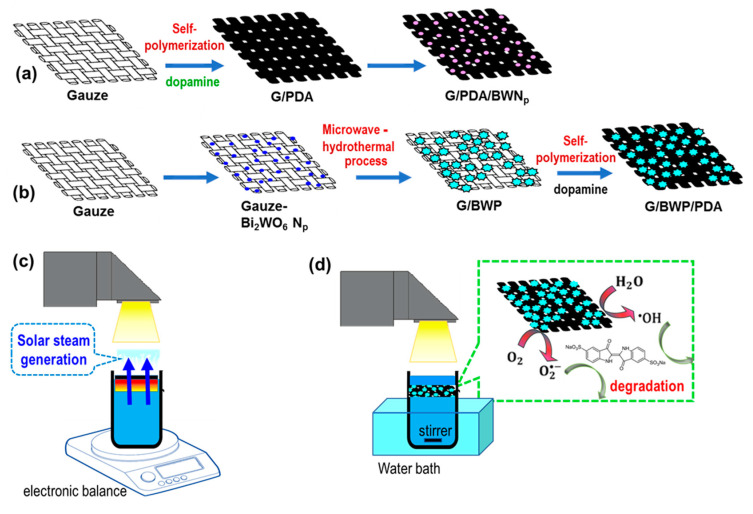
Procedures of the formation of Bi_2_WO_6_-polydopamine decorated gauze (**a**) G/PDA/BWNp (gauze/polydopamine/Bi_2_WO_6_ nanoparticles) (**b**) G/BWP/PDA (gauze/Bi_2_WO_6_ nanoplates/polydopamine) samples, schematic illustrations of the setup for (**c**) solar steam generation test (**d**) photocatalytic degradation test.

For the first type of sample, polydopamine was grown on the gauze substrate by self-polymerization. A dopamine solution was prepared by dissolving 0.25 g of dopamine hydrochloride in 50 mL of deionized water. Then, a 2 M NaOH solution was used to tune the pH value of the solution to 8.5. The gauze substrate (2 cm × 2 cm) was immersed in the dopamine solution at room temperature for 24 h to make the G/PDA sample. Then, a uniform Bi_2_WO_6_ nanoparticle layer was formed by the alternative immersion of the G/PDA sample into separately placed Bi(NO_3_)_3_ (0.06 M, 50 mL) and Na_2_WO_4_ (0.06 M, 50 mL) solution for 3 min. Before immersion in another solution, the gauze-based sample was immersed in D.I. water for 1 min. The alternative immersion was repeated 10 times. Then, the G/PDA/BWNp sample was obtained after washing by water and drying at 60 °C for 24 h (Figure 1a).

For the second type of sample, G/BWP/PDA was prepared by the consecutive growth of Bi_2_WO_6_ nanomaterials and PDA on gauze substrates (Figure 1b). The Bi_2_WO_6_ nanomaterials were grown by a two-step process. At first, a uniform Bi_2_WO_6_ nanoparticle layer was formed on the gauze by the alternate immersion of the gauze substrate (2 cm × 2 cm) into separately placed Bi(NO_3_)_3_ (0.06 M, 50 mL) and Na_2_WO_4_ (0.06 M, 50 mL) solution. Before the immersion in another solution, the gauze-based sample was rinsed with D.I. water for 1 min. The alternative immersion was repeated ten times. G/BWNp sample was obtained after washing by water and drying at 60 °C for 24 h. For the second step, Bi_2_WO_6_ nanoplates (BWP) were grown on the previous G/BWNp substrate to make the G/BWP sample by a microwave-assisted hydrothermal method. 0.365 g of Bi(NO_3_)_3_ was dissolved in 20 mL of ethylene glycol to prepare solution A. 0.125 g of Na_2_WO_4_ was dissolved in 20 mL of ethylene glycol to prepare solution B. The G/BWNp substrate (2 cm × 2 cm), solution A, and solution B were put in a EasyPrep Plus^®^ closed vessels (100 mL). The reaction mixture was sealed and heated by a Microwave Synthesis System (MARS 6, CEM, NC, USA) at 160 °C for 20 min to make the G/BWP sample. Then, polydopamine was grown on the G/BWP by self-polymerization to make the G/BWP/PDA sample. A dopamine solution was prepared by dissolving 0.25 g of dopamine hydrochloride in 50 mL of deionized water. Then, a 2 M NaOH solution was used to tune the pH value of the solution to 8.5. The G/BWP sample (2 cm × 2 cm) was immersed in the prepared dopamine solution for 24 h at room temperature. Then, the G/BWP/PDA sample can be obtained after being washed with deionized water three times and dried at 60 °C (Figure 1b).

### 2.3. Solar Steam Generation

The solar steam generation test was performed to investigate the light-to-heat conversion performance of various samples (Figure 1c). A fluoro acrylate copolymer-based water repellent agent solution (JR7101, J-Young Technology Corp., Hsinchu city, Taiwan) was spray-coated on the top layer of the sample (2 cm × 2 cm) and dried at 60 °C for 10 min to make the sample floatable. In the solar steam generation experiment, 20 mL of water was added to a beaker with a diameter of 2.9 cm (height 4.1 cm), and the sample was floated on the water. The light intensity of the simulated solar light (Xenon lamp, PX350A, Prosper OptoElectronic Co., New Taipei City, Taiwan) was tuned at 1000 W/m^2^. The thermal images and surface temperature of the membranes floating on the solution were monitored by an infrared thermal imaging camera (FLIR-A320, FLIR SYSTEMS, Wilsonville, OR, USA). An analytical balance (GF2000, A&D, Tokyo, Japan) connected to a computer was used for real-time monitoring of the mass changes during the photothermal evaporation test. The whole evaporation process was carried out under the ambient temperature of 30 °C and relative humidity of 70%.

### 2.4. Photocatalytic Degradation

The photocatalytic activities were evaluated for the decoloration of the indigo carmine solution under light irradiation. The photocatalytic degradation of indigo carmine dye was performed in a reactor using 10 mL of a 60 ppm dye solution and the photocatalysts (2 cm × 2 cm). Before the irradiation, the solution was magnetically stirred for 60 min in the dark to reach an absorption-desorption equilibrium. These solutions were illuminated with a 350 W Xe lamp (Prosper HID). The temperature of the solution was maintained at 30 °C using a water bath. The dye degradation was monitored by a JASCO V-770 UV-vis spectrophotometer.

### 2.5. Characterization

The morphologies of samples were analyzed with a field scanning electron microscope (FESEM, HITACHI, S-4800) and a field-emission transmission electron microscope (TEM, JEOL, JEM-2100F). The surface chemical composition and elemental valence were identified from X-ray photoelectron spectra (XPS, ULVAC-PHI, PHI 5000 Versa Probe), using C 1s peak as a reference to calibrate binding energies. The surface wettability of the samples was evaluated with a contact-angle meter (CAM-100, Creating-Nanotech Co., Tainan city, Taiwan). The Raman spectrum was measured by a Raman microscope (MRI532S, Protrustech, Tainan city, Taiwan) with an emission wavelength of 532 nm (He–Ne laser). The detector integration time and laser irradiation power were 10 s and 2 mW. The diffuse reflectance spectra (DRS) were recorded by a spectrometer (JASCO V-770, Tokyo, Japan) to measure the light absorption property of photocatalysts.

## 3. Results and Discussion

### 3.1. Morphology

**Figure 2:**
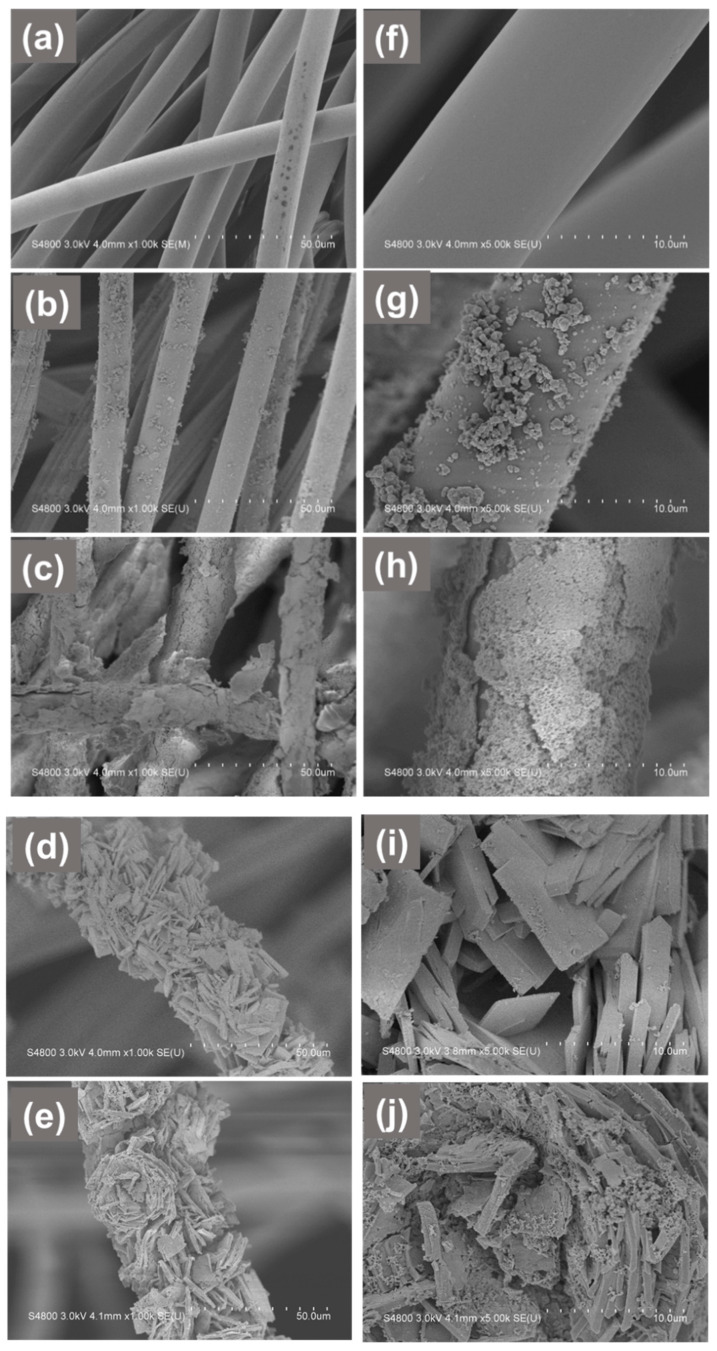
Field-emission scanning electron microscopy (FESEM) images of (**a**,**f**) gauze (**b**,**g**) G/PDA (**c**,**h**) G/PDA/BWNp (**d**,**i**) G/BWP (**e**,**j**) G/BWP/PDA.

The fiber surface morphology of the membrane changed after the growth of Bi_2_WO_6_ nanoplates and polydopamine on the surface of gauze, as observed by the FESEM images. Figure 2 illustrates the FESEM images of gauze, G/PDA, G/PDA/BWNp, G/BWP, and G/BWP/PDA samples. As shown in Figure 2a,f, the fibers of pristine gauze show a smooth surface with a diameter ranging between 9 to 11 μm. The polydopamine was grown on the gauze substrate by self-polymerization to enable the G/PDA sample to act as a comparative sample. Parts of the surfaces of PDA-modified fibers for G/PDA are covered by some particles with the size ranging from 150 nm to 500 nm, due to the polymerization of dopamine (Figure 2b,g). Then, Bi_2_WO_6_ nanoparticles (BWNp) were grown on the surface of G/PDA by a successive ionic layer adsorption and reaction process to prepare G/PDA/BWNp sample. As shown in Figure 2c,h, BWNp with the size of less than 100 nm was uniformly distributed on almost all the fiber surfaces of G/PDA/BWNp. The Bi_2_WO_6_ nanomaterials were grown by a two-step process to achieve uniform modification of the fiber surface. The first step was a successive ionic layer adsorption and reaction route. The second step was a microwave-assisted hydrothermal process. The morphology of the G/BWP sample (Bi_2_WO_6_ nanoplate modified gauze) is shown in Figure 2d,i. For the G/BWP sample, the surface of the fiber is fully covered with a lot of assembled two-dimensional Bi_2_WO_6_ nanoplates. The enlarged image of the G/BWP sample (Figure 2i) shows that Bi_2_WO_6_ grows into two-dimension rectangular-plate-like morphologies with a side length of 5–10 μm and the width of 1.0–5.5 μm. The thickness of an individual Bi_2_WO_6_ nanoplate is 0.22–1.1 μm. Bi_2_WO_6_ nanoplates were randomly stacked together. Besides, the fiber diameter of G/BWP increases, ranging between 35 to 55 μm (Figure 2d). The increased fiber diameter results from the formation of large amounts of nanoplates on the fiber surface. Then, polydopamine was grown on the surface of G/BWP to make the G/BWP/PDA sample. Figure 2e,j show that randomly stacked Bi_2_WO_6_ nanoplates were still observed on G/BWP/PDA. Besides, compared to the enlarged image of G/BWP (Figure 2i), there are lots of PDA nanoparticles formed on the Bi_2_WO_6_ nanoplates (Figure 2j). The fiber diameter of G/BWP/PDA (Figure 2d) is close to G/BWP (Figure 2e).

### 3.2. Chemical Compositions

#### 3.2.1. Transmission Electron Microscopy Energy-Dispersive X-ray (TEM EDX)

**Figure 3:**
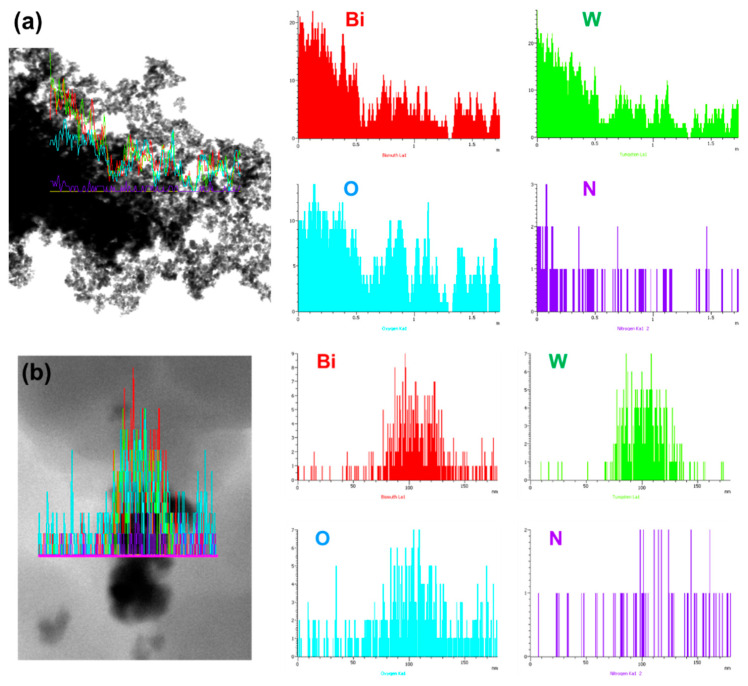
Transmission electron microscopy (TEM) image and energy-dispersive X-ray (EDX) spectrum of line scan of (**a**) G/BWP/PDA (**b**) G/PDA/BWNp.

The TEM energy-dispersive X-ray (EDX) spectrum of a line scan is presented in Figure 3a to investigate the elemental distribution for the powder scratched from the G/BWP/PDA composite sample. The Bi, W, O, and N elements are found in the G/BWP/PDA sample. The elements of Bi, W, and O indicate the existence of Bi_2_WO_6_. The N element originates from dopamine due to the successful polymerization of dopamine on the fabrics. The signals of the Bi, W, O, and N elements are higher near the center of the line, indicating that the plate-like sample is Bi_2_WO_6_, and polydopamine is distributed well near Bi_2_WO_6_. Similar results were observed for the powder scratched from the G/PDA/BWNp composite sample (Figure 3b).

#### 3.2.2. X-ray Photoelectron Spectra (XPS) Analysis

**Figure 4:**
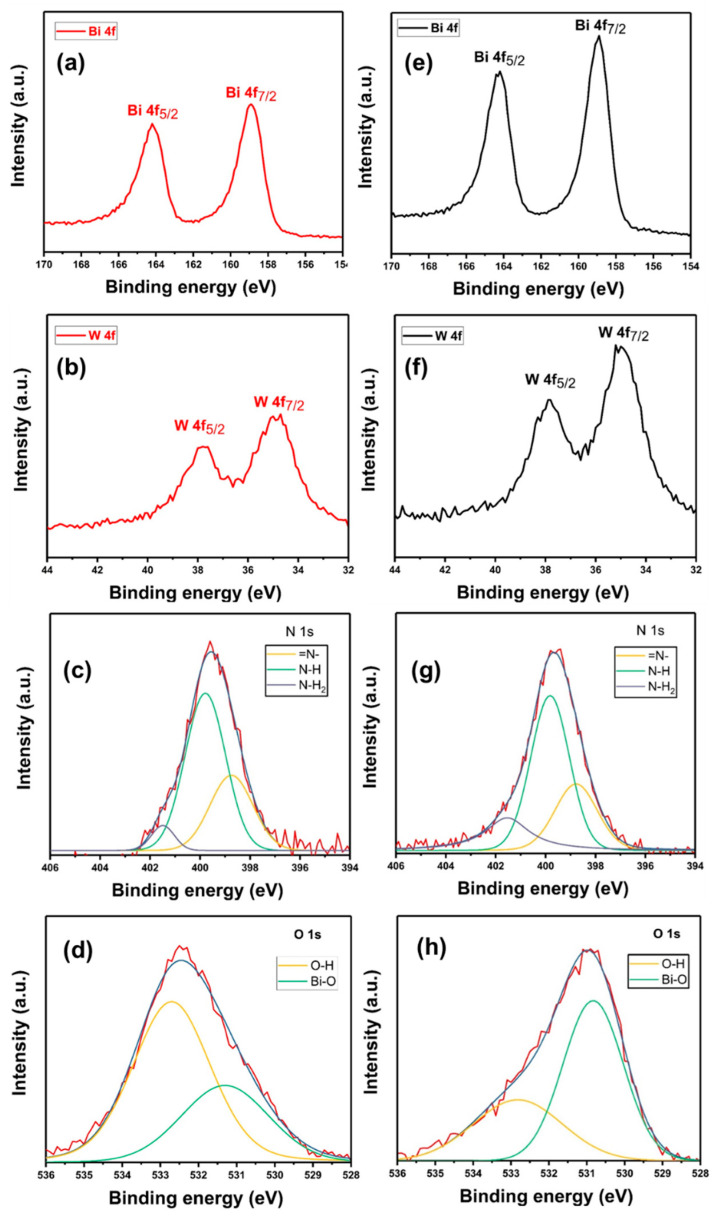
X-ray photoelectron spectra (XPS) (**a**) Bi 4f (**b**) W 4f (**c**) N 1s (**d**) O 1s of G/BWP/PDA and XPS (**e**) Bi 4f (**f**) W 4f (**g**) N 1s (**h**) O 1s spectra of G/PDA/BWNp.

The XPS analysis of G/BWP/PDA sample was measured to analyze its oxidation state and chemical composition. Figure 4 shows the XPS (a) Bi 4f (b) W 4f (c) N 1s (d) O 1s spectra of G/BWP/PDA. The Bi 4f peaks of G/BWP/PDA at 158.9 and 164.2 eV can be assigned to Bi 4f_7/2_ and Bi 4f_5/2_, indicating the Bi^3+^ of Bi_2_WO_6_. The W 4f peaks of G/BWP/PDA at 37.8 and 34.7 eV are attributed to W 4f_5/2_ and W 4f_7/2_, respectively, which are related to W^6+^ of Bi_2_WO_6_ [61,62]. The N 1s peak is deconvoluted into three peaks (Figure 4c). That peaks at 401.5, 399.8, and 398.7 eV can be attributed to primary amine (R–NH_2_), secondary amine (R1-NH-R2), and tertiary amine (=N-R) groups, respectively [63,64]. The results are related to the chemical structures of polydopamine, possible intermediate species, and dopamine monomer [65]. The primary amine is related to dopamine. The secondary amine can be assigned to the intermediate species or polydopamine, while the tertiary amine can be attributed to tautomeric species of the intermediates. The O 1s spectrum of G/BWP/PDA was deconvoluted into two peaks. The peaks at 531.3 and 532.7 eV are assigned to Bi–O, and O–H, respectively. These results support the formation of Bi_2_WO_6_ and polydopamine on the sample. Figure 4e–h present the XPS Bi 4f, W 4f, N 1s, and O 1s spectra of G/PDA/BWNp, respectively. The results of the G/PDA/BWNp sample were similar to those of G/BWP/PDA, except that the Bi–O peak was higher than the O–H peak in the O 1s spectrum. 

#### 3.2.3. Raman Spectra

**Figure 5:**
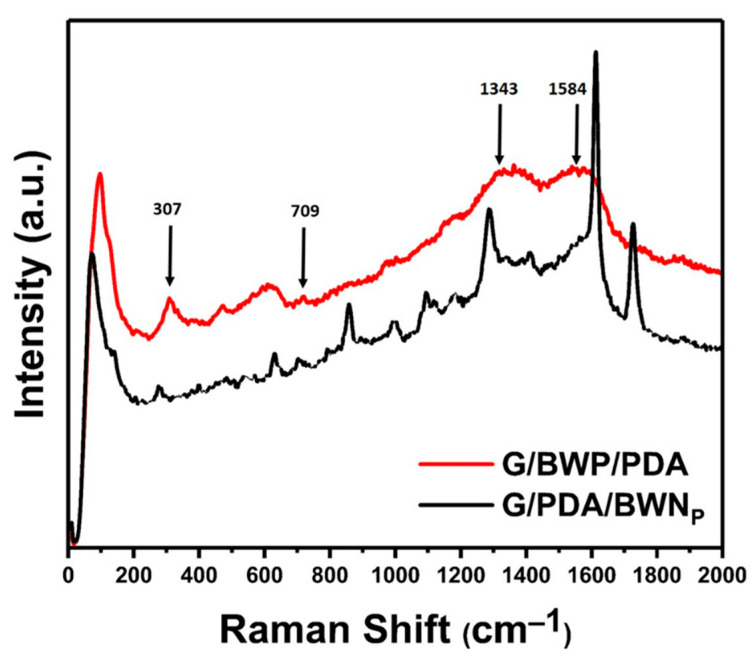
Raman spectra of G/BWP/PDA and G/PDA/BWN_P_.

The surface chemistry of G/BWP/PDA and G/PDA/BWN_P_ was analyzed by the Raman spectra (Figure 5). The peak at 709 cm^−1^ is associated with the asymmetric stretching mode of WO_6_ octahedra for the vibrations of equatorial oxygen atoms within layers. The peak at 307 cm^−1^ was related to the simultaneous translational movement of Bi^3+^ and the bending of WO_6_ octahedra [66,67]. Besides, two broad peaks at 1343 and 1584 cm^−1^ were attributed to the catechol stretching vibration and deformation from the chains of polydopamine. Similar results were reported in the literature [68,69,70,71]. These peaks support the existence of polydopamine and Bi_2_WO_6_ on G/BWP/PDA sample. Similar peaks were found for the G/PDA/BWN_P_ sample.

### 3.3. Diffuse Reflection Spectra (DRS)

**Figure 6:**
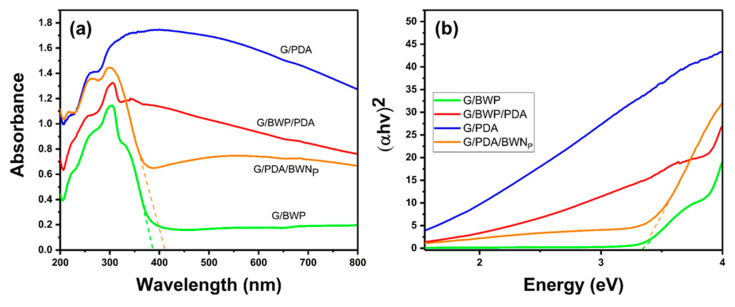
(**a**) Diffuse reflection spectra (DRS) (**b**) Tauc plot of G/BWP, G/BWP/PDA, G/PDA, and G/PDA/BWN_P_.

The light absorption properties of various samples were observed by a diffuse reflection spectrophotometer. G/BWP samples (Figure 6a) showed a strong light absorption in the UV region ranging from 200 to 380 nm. Compared with the G/BWP samples, G/BWP/PDA exhibits an increase in UV and visible light absorption ranging from 200 to 800 nm. The G/PDA membrane exhibits the highest UV and visible light absorption among the four samples. After the decoration of BWN_p_ on the G/PDA, the G/PDA/BWN_P_ sample showed decreased light absorption than the G/PDA membrane. The results of G/BWP/PDA and G/PDA/BWN_P_ exhibit the influences of the spatial distribution of Bi_2_WO_6_ and polydopamine on the light absorption properties of G/BWP/PDA and G/PDA/BWN_P_. The composition that appeared on the top layer exhibits a greater influence on the light absorption of the composite membrane. G/BWP/PDA with polydopamine on the top layer show larger absorption in the visible light region, while the G/PDA/BWN_P_ sample has higher absorption in the UV light range. Figure 6b presents the Tauc plots of G/BWP, G/BWP/PDA, G/PDA, and G/PDA/BWN_P_. The bandgaps of two types of Bi_2_WO_6_ (BWP and BWN_p_) were obtained from G/BWP and G/PDA/BWN_P_ samples because the Bi_2_WO_6_ nanomaterials were located on their top layer. The bandgaps of BWP and BWN_p_ were 3.33 and 3.35 eV, respectively.

### 3.4. X-ray Diffraction (XRD) Spectra

**Figure 7:**
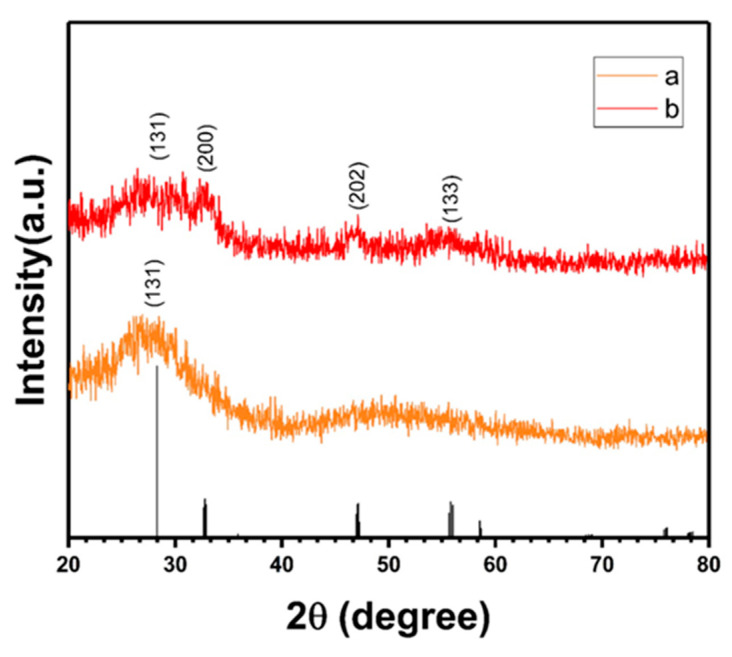
X-ray diffraction (XRD) spectra of (**a**) G/PDA/BWN_P_ (**b**) G/BWP/PDA.

Figure 7 presents the XRD spectra of G/PDA/BWN_P_ and G/BWP/PDA. A broad diffraction peak of G/PDA/BWN_P_ photocatalyst at 2θ = 28.4° is assigned to the (131) plane of the orthorhombic phase of Bi_2_WO_6_ (JCPDS#79-2381). There is a wide peak at around 2θ = 50°. The broad XRD diffraction peaks indicated weak crystallinity of the G/PDA/BWN_P_ photocatalyst. The main characteristic diffraction peaks of the G/BWP/PDA photocatalyst observed at 2θ = 28.4°, 32.8°, 47.2°, and 55.7° are attributed to the (131), (200), (202), and (133) planes of the orthorhombic phase of Bi_2_WO_6_ (JCPDS#79-2381), respectively. These were consistent with the results reported in other literature [72,73], indicating the formation of Bi_2_WO_6_ in G/BWP/PDA.

### 3.5. Surface Hydrophilicity

**Figure 8:**
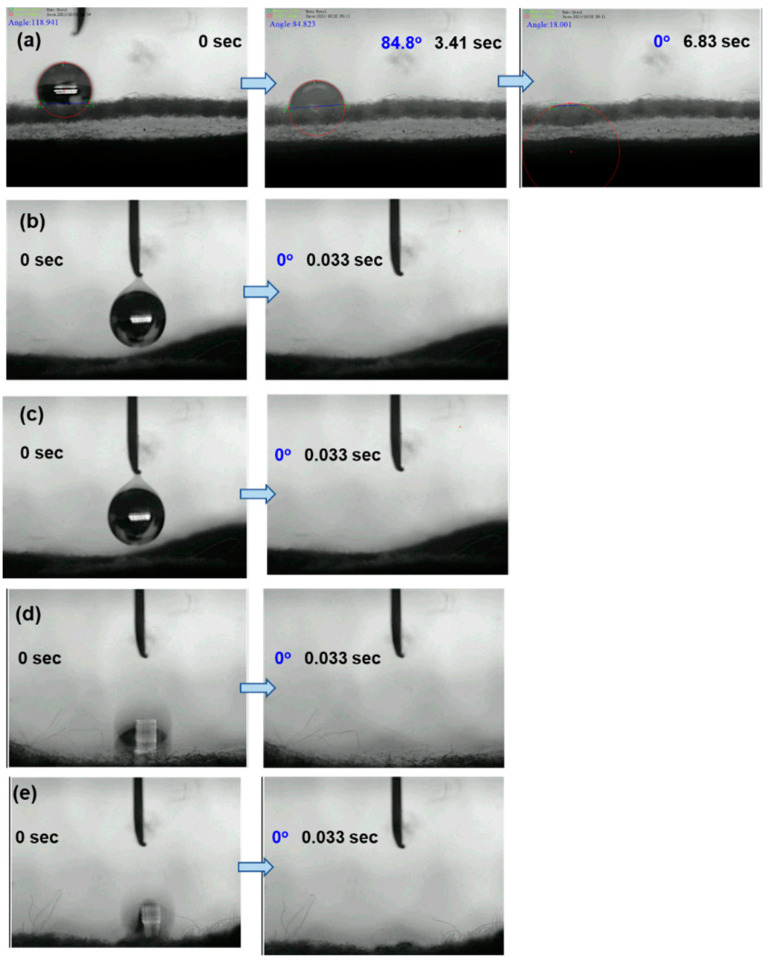
Dynamic contact angles of water droplets on (**a**) gauze (**b**) G/PDA (**c**) G/BWP (**d**) G/BWP/PDA (**e**) G/PDA/BWN_P_.

An efficient water supply is essential to the preparation of an effective solar steam generation membrane. In this study, the SEM images of modified samples revealed that the porous structures of gauze substrates remained after the growth of polydopamine and Bi_2_WO_6_ nanomaterials. These encapsulated materials should be hydrophilic to facilitate water supply and steam escape. The surface hydrophilicity also facilitates the contact between the photocatalyst and the organic pollutant. The surface hydrophilicity was evaluated by measuring the dynamic contact angles of water droplets on different samples (without coating the hydrophobic layer) to elucidate their potential applications in solar evaporation and photocatalytic degradation. As shown in Figure 8a, the complete wetting of a water droplet on pristine gauze was achieved within 6.83 s. However, G/PDA (Figure 8b), G/BWP (Figure 8c), G/BWP/PDA (Figure 8d), and G/PDA/BWN_P_ (Figure 8e) samples exhibited rapid, complete wetting within 0.033 s. The surface hydrophilicity was further enhanced after the loading of polydopamine and Bi_2_WO_6_ nanomaterials. 

### 3.6. Photocatalytic Property

**Figure 9:**
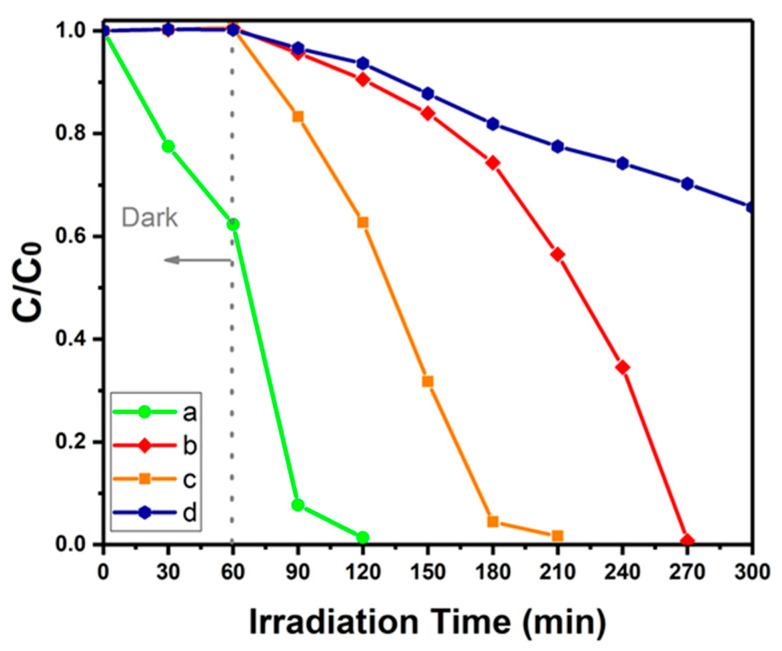
Photodegradation of indigo carmine (60 ppm) under circulating water conditions by (**a**) G/BWP (**b**) G/BWP/PDA (**c**) G/PDA/BWN_P_ (**d**) without photocatalyst (blank).

The activity of different immobilized photocatalysts (G/BWN_P_, G/BWP, G/BWP/PDA) under light illumination was investigated through photocatalytic decoloration experiments using indigo carmine (IC) as the pollutant model compound. The photocatalytic degradation performance of various samples is shown in Figure 8. The initial concentration of indigo carmine (60 ppm) and the size of the membrane (2 cm × 2 cm) were kept the same in all experiments for the comparison of different samples. For the Bi_2_WO_6_ nanoplates-based G/BWP sample, 39% of IC was adsorbed on the photocatalyst during the dark test (Figure 9a). The absorption peak of residual IC solution at 610 nm decreases rapidly under light irradiation, indicating the high photocatalytic degradation activity of G/BWP. 99% of IC can be degraded after the irradiation for 60 min in the presence of G/BWP. However, the IC adsorption property changed when PDA was grown on G/BWP. No noticeable decrease of IC was observed for the dark test of the G/BWP/PDA sample, indicating the adsorption of IC on the G/BWP/PDA was negligible (Figure 9b). Besides, the photocatalytic degradation activity of G/BWP/PDA was lower than that of G/BWP. The residual concentration of IC solution decreases gradually after light irradiation. The irradiation time required for degradation of 99% IC by G/BWP/PDA is 210 min. Such a decrease in the photocatalytic activity of the G/BWP/PDA sample may result from the coverage of B_2_WO_6_ nanoplates by polydopamine, leading to a reduction in the exposed active sites of B_2_WO_6_ nanoplates. Such a problem can be solved by changing the formation sequence of Bi_2_WO_6_ and polydopamine on the gauze substrate. The G/PDA/BWN_P_ photocatalyst was prepared by the deposition of polydopamine on the gauze substrate by self-polymerization, followed by the formation of a uniform Bi_2_WO_6_ nanoparticle layer on top. Compared with G/BWP/PDA, the photocatalytic activity of the G/PDA/BWN_P_ membrane was improved. Degradation of 96% of IC was achieved after being irradiated for 120 min in the presence of G/PDA/BWN_P_ (Figure 8c). Photodegradation of indigo carmine (60 ppm) without photocatalyst was shown in Figure 9d as a blank test. Its decoloration rate was slower than other three curves with different photocatalysts. De Andrade et al. [74] studied the degradation of the indigo carmine dye in aqueous medium by the autoclaved cellular concrete/Fe_2_O_3_ catalyst in Fenton-like and photocatalytic processes. The toxicity tests against Vero cells indicated that the toxicity of the degradation products, generated by both processes, is smaller or similar to the precursor dye. Vautier et al. [56] reported the photocatalytic degradation of indigo carmine using UV-irradiated titania-based catalysts. The oxidative agents are photo-produced holes h^+^ and/or ●OH radicals. A detailed degradation pathway, based on careful identification of intermediate products, is proposed. In addition to a prompt removal of the color, photocatalysis can mineralize carbon and of nitrogen and sulfur heteroatoms into innocuous compounds. In our previous study [53], the photocatalytic degradation by Ag/flower-like Bi_2_WO_6_ photocatalysts with and without scavengers reveals that h^+^ and ●O_2_^−^ are the major active species generated by the photocatalyst. The oxidation of water by holes can generate ●OH. We believe the products of the photocatalytic degradation using Bi_2_WO_6_ photocatalysts will not be more toxic than the parent compound, indigo carmine.

### 3.7. Solar-Steam Generation

**Figure 10:**
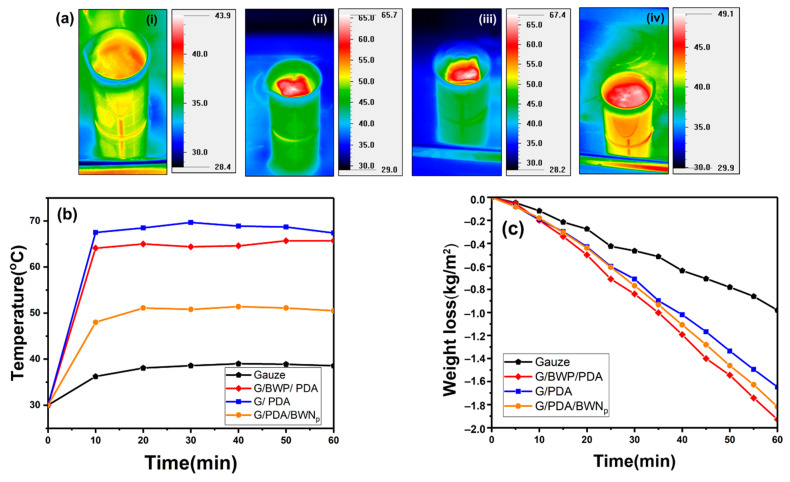
(**a**) Bulk water temperature distribution of (i) gauze (ii) G/BWP/PDA (iii) G/PDA (iv) G/PDA/BWN_P_, and (**b**) surface temperature profiles over time (**c**) mass loss of water over time by gauze, G/PDA, G/BWP/PDA, G/PDA/BWN_P_ after simulated solar illumination at 1 kW m^−2^ (1 sun).

The photothermal and solar steam generation performance of various samples (G/PDA, G/BWP/PDA, gauze) were evaluated. Typical infrared thermal images and the surface temperature changes of different membranes recorded by an infrared camera are shown in Figure 10a,b, respectively. Figure 10a reveals that the temperatures around the floated composite films are all higher than the bulk solution. Figure 10b shows the surface temperature profiles of G/PDA, G/BWP/PDA, G/PDA/BWN_P_, and gauze samples versus time under the simulated solar illumination. Before the light illumination, the initial surface temperature of all samples is about 30 °C. After the simulated solar illumination with a power density of 1000 W m^−2^ for 1 h, the surface temperature of the gauze is 36.1 °C. However, the surface temperatures of G/PDA and G/BWP/PDA samples reached 67.4 and 65.7 °C, respectively. These two samples with PDA on the top layer showed similar surface temperatures. The Bi_2_WO_6_ nanoplates under the PDA polymer did not deteriorate the light-to-heat transform property of polydopamine. Han et al. [75] studied the dual functional polydopamine-modified CuS@HKUST for quick sterilization through enhanced photothermal and photocatalytic ability. They found that the nanoparticles’ ability to produce heat was improved after the coating of polydopamine. Compared with G/BWP/PDA, the G/PDA membrane exhibits higher light absorption (Figure 6) and photothermal-induced temperature rise (Figure 10a). The surface temperature of G/PDA/BWN_P_ (49.0 °C) is lower than that of G/PDA. The Bi_2_WO_6_ nanomaterials exhibit lower light absorption than PDA (Figure 6). PDA also has excellent photothermal conversion properties. It may explain why the coverage of polydopamine by B_2_WO_6_ nanoparticles leads to the decrease of surface temperature from 67.4 (G/PDA) to 49.0 °C (G/PDA/BWN_P_). The distribution of PDA and Bi_2_WO_6_ on the samples has some influences on the temperature increase. G/BWP/PDA with PDA-coated G/BWP structure exhibit a higher temperature increase than the G/PDA/BWN_P_ sample with BWN_P_-coated G/PDA structure. The surface temperatures reached the maximum values for all samples after 10 min of irradiation (Figure 10b). The photothermal-induced temperature rise of gauze (as a comparative sample) was much lower than G/PDA and G/BWP/PDA. The results indicate the high light absorption and effective photothermal conversion properties of these PDA-based samples (G/PDA, G/BWP/PDA, G/PDA/BWN_P_). Meanwhile, polydopamine is the critical component for the photothermal conversion properties of composite materials. Figure 10c presents the mass loss of water over time by G/PDA, G/BWP/PDA, gauze after simulated solar illumination at 1 kW m^−2^. The water evaporation rates of the Bi_2_WO_6_-PDA based films did not follow the trend of the light-induced temperature rise. The water evaporation rates of the G/BWP/PDA sample under the irradiation of the Xe lamp (light intensity = 1000 W/m^2^) reached 1.94 kg·m^−2^·h^−1^. Although the G/PDA and G/BWP/PDA films exhibited similar light-induced temperature rise, G/BWP/PDA showed a higher water evaporation rate than the G/PDA film (1.68 kg·m^−2^·h^−1^). The water evaporation rates of the functional films depend on the photothermal effect of the material, film structure for water supply and steam escape, and thermal management [76]. As shown in the SEM images (Figure 2), there are interstices among the Bi_2_WO_6_ nanoplates for G/BWP/PDA. Compared with the G/PDA film, the G/BWP/PDA sample with Bi_2_WO_6_ nanoplate aggregates on the fiber surface may provide a better capillary structure for water supply and steam escape. Besides, both the G/PDA and G/BWP/PDA films showed higher water evaporation rates than the gauze (0.93 kg m^−2^ h^−1^). The G/PDA/BWN_P_ membrane shows a higher water evaporation rate (1.83 kg m^−2^ h^−1^) than the G/PDA sample (Figure 10c). The Bi_2_WO_6_ nanomaterials may contribute to water transport and steam escape. These photothermal membranes can convert light energy into localized heat and vaporize water across the microporous surface into steam.

## 4. Conclusions

A Bi_2_WO_6_-polydopamine (PDA) modified gauze was developed as a dual-functional membrane for application in photocatalytic degradation and solar steam generation. The spatial distribution of Bi_2_WO_6_ and polydopamine has a great influence on the light absorption properties, photocatalytic degradation, and solar steam generation performances of G/BWP/PDA and G/PDA/BWN_P_. G/BWP/PDA with polydopamine on the top layer showing a higher photothermal temperature rise. G/PDA/BWN_P_ with Bi_2_WO_6_ nanoparticles on top exhibit higher photocatalytic activity and similar solar steam generation performance when compared with G/BWP/PDA. Active sites of Bi_2_WO_6_ nanoparticles that were not covered by polydopamine may contribute to the improved photocatalytic performance of G/PDA/BWN_P_. For the G/BWP/PDA and G/PDA/BWN_P_ with better solar steam generation performance than G/PDA, the Bi_2_WO_6_ nanomaterials may contribute to the water transport and steam escape. The membrane with an interconnected pore structure can facilitate the contact between Bi_2_WO_6_ and dye pollutants, and the capillary transport of water to the top surface. Degradation of 96% of IC was achieved after irradiation for 120 min in the presence of G/PDA/BWN_P_. The photothermal water evaporation rate and surface temperature of the G/BWP/PDA membrane under irradiation (1 sun) reached 1.94 kg m^−2^ h^−1^ and 65.7 °C, respectively. For the application of water production in remote areas without electricity supply, a dual-functional membrane (G/BWP/PDA or G/PDA/BWPNp) using a ternary compound may be a good choice. People can collect clean water by condensing the solar-generated water vapor, and can degrade the pollutant before it flows into the water body.

## Data Availability

The data presented in this study are available on request from the corresponding author.
